# Antibacterial Activity and Toxicity of Analogs of Scorpion Venom IsCT Peptides

**DOI:** 10.3390/antibiotics6030013

**Published:** 2017-06-28

**Authors:** Roberto de la Salud Bea, Adam F. Petraglia, Michael R. Ascuitto, Quentin M. Buck

**Affiliations:** Department of Chemistry, Rhodes College, 2000 North Parkway, Memphis, TN 38112, USA; adam.petraglia@gmail.com (A.F.P.); mrascuitto@yahoo.com (M.R.A.); Quentin.Buck@bcm.edu (Q.M.B.)

**Keywords:** antimicrobial peptides, α-Helix, amphipathic, hemolysis

## Abstract

Seven analogs of the natural, α-helix peptides IsCT1 and IsCT2—found in the venom of scorpion *Opithancatus Madagascariensis*—have been synthesized and tested to compare their antibacterial and hemolytic activity against natural peptides. In general, results show that increasing hydrophobicity by substituting positions 5 and 9 of the sequences with alanine, valine, and leucine, enhances antibacterial activity. However, this also increases hemolytic activity. The analog with an increased net positive charge from +1 to +3 produces moderate bacterial growth inhibition but also has high hemolytic activity. On the other hand, the analog with a negative net charge (−1) has low antibacterial properties but also no cytotoxicity under the tested conditions, a similar result was found for five of the seven studied analogs.

## 1. Introduction

In recent years, pathogenic organisms have developed resistance to highly utilized drugs, not only for humans, but also for animals [1]. This increasingly renders traditional antibiotic treatments ineffective. Thus, it is necessary to find new treatments for these resistant diseases [2,3,4]. A new interest in natural products has produced the discovery of non-traditional compounds, such as venoms, with valuable medicinal properties used as anticoagulants, treatment for diabetes, or analgesics [5,6]. Antimicrobial peptides are typically short length peptides (5 to 100 amino acids) found in blood, immune system cell secretions, and venoms of animals or plants [7,8,9]. These peptides can adopt several secondary structures, including α-helices and beta sheets with or without cysteines and disulfide bonds. However, all of them have in common the presence of a hydrophobic side with hydrophobic amino acids and a hydrophilic side containing cationic amino acids. Although these peptides can have different lengths and structures, their amphipathic properties are essential for their mechanism of action. The most commonly accepted mechanism suggests the peptides bind the cell surface create distortions that are followed by the formation of pores or the complete destruction of the cell membrane and the release of plasma contents [10]. New studies have also proposed mechanisms involving direct interaction with intracellular targets [11].

Venoms are complex mixtures of components used by animals and plants for defense or to attack enemies or prey. IsCT1 (ILGKIWEGIKSLF) and IsCT2 (IFGAIWNGIKSLF) (Isalo CytoToxic) are two peptides found by Dai in the venom of scorpion *Opithancatus madagascariensis* [12]. These two 13 amino acid long peptides have an amidated C-terminus and form an α-helix secondary structure with amphipathic properties. Studies show significant antimicrobial activity against Gram-positive and Gram-negative bacteria but, as expected by their poisonous origin, they also have high hemolytic activity that prevents clinical practicality [13]. Nevertheless, several analogs with substituted residues in defined positions of the sequence show promising antimicrobial activity with moderate or minimal toxicity [14]. These compounds have the mechanism of action of other amphipathic peptides. However, in a recent paper, Ghosh also suggests that some IsCT analogs may have activity without disrupting the cell membrane by penetrating it and inhibiting bacterial macromolecule biosynthesis, such as nucleic acids and proteins [15].

In our group, we have synthesized seven analogs of the two natural IsCT1 and IsCT2 peptides and tested their antibacterial and toxic (hemolytic) activities. Modifications have been made on both the hydrophobic and the hydrophilic sides of the helix. On the hydrophobic side (positions 5 and 9 of the sequence) we have substituted alanine, valine, and leucine in order to test how an increase in amino acid size, and in this way, through peptide volume and hydrophobicity, affect antibacterial activity. On the hydrophilic side (positions 7 and 10 of the sequence) we have synthesized a lysine substituted analog, IsCT1K7 [16] to introduce positive charges and also a glutamic acid substituted analog, IsCT1E7 to introduce negative charges under experimental conditions.

## 2. Results

### 2.1. Peptide Design IsCT

Seven peptides analogs were synthesized using two naturally found 13-residue, amphipathic peptides IsCT1 and IsCT2 as reference models (Table 1). These natural peptides have a free N-terminal and an amidated C-terminus with no cysteine (thus, with no disulfide bonds) and adopt an α-helix conformation in hydrophobic environments.

**Figure 1:**
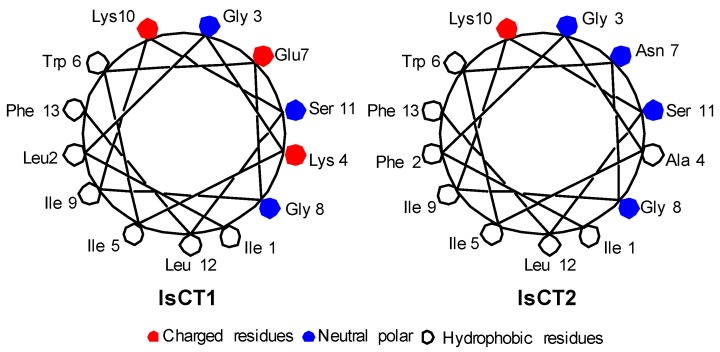
Schiffer and Edmunson alpha helix wheel projection of IsCT1 and IsCT2 peptides.

To test the influence of increasing residue size and hydrophobicity in the structure and activity of the analogs, positions 5 and 9 on the hydrophobic side of the helix (Figure 1) were systematically substituted with the hydrophobic amino acids alanine, valine, and leucine. For IsCT1 K7 analog, the substitution of a lysine on position 5 increases total charge from +1 to +3. Although IsCT1K7 was first synthesized by Kim [16], we used it in this work in order to compare its activity under our experimental conditions against the other analogs and to specifically test the influence of positive and negative charged amino acids on overall activity. Thus, we also made substitutions of the same hydrophilic positions (7 and 10) with glutamic acid to switch the total charge to −1 for peptide IsCTE7. The original natural peptides were also synthesized for reference and comparison.

Based on the model for the α-helix structure for these peptides, it can be seen in these star projections (Figure 1) for IsCT1 and IsCT2 that positions 5 and 9 fall on a hydrophobic section of the peptide while positions 7 and 10 are on the hydrophilic side.

### 2.2. Secondary Structure

Our main goal was not to study full mechanism of action or physical interaction of our peptides with cell membranes. However, to have an idea of what type of secondary structure our peptides were able to form, we dissolved them in water and in a solution of the helix promoter trifluoroethanol (TFE) in water. Circular Dichroism (CD) analysis shows peptides with a random structure in water (Table 2 and Figure S1). On the other hand, all peptides show a helical structure in the hydrophobic environment 50% TFE in water. Although some peptides were tested in 70% TFE, the results were not different to 50% TFE. In general, our IsCT peptide analogs show a helical structure, though lower to equally substituted analogs of similar BmKn peptides, also extracted from scorpion venoms [17]. Interestingly, the two alanine analogs (IsCT1A1 and 2A1) had the lowest helical structure despite the reported α-helix stabilizing properties of this amino acid [18].

This was also observed for BmKn peptides [19]. It is also apparent that an increase in size of the substituted amino acid (from alanine to valine to leucine), increases the percent helix. It is likely that hydrophobicity is the major factor for this observation and the total increase in peptide volume does not interfere with the formation of a more organized secondary structure. Also, the glutamic acid substituted analog has the highest percent helix, suggesting that altering the net charge from +1 to −1 does not perturb the peptide structure either.

### 2.3. Antimicrobial Activities

Antimicrobial activities were determined by standard broth dilution against two Gram-positive and three Gram-negative bacteria. In general, the MICs seem to be higher than the concentrations tested in our experiments (Table 3). Only *Staphylococcus aureus* and *Escherichia coli* seem to be inhibited with both natural peptides but also by the leucine substituted analogue, IsCT1L1, at concentrations lower than 25 μg/mL. The lysine substituted analogue (IsCT1K7) with two extra positive charges (+3) seems to be more effective than other analogs with a +1 charge. The negatively charged (−1) peptide (IsCT1E7), although having the highest percentage α-helix structure, does not show the expected inhibition, with higher than 100 μg/mL for all tested bacteria. Curiously, some peptides—such as IsCT1V1, IsCT1E7, or even natural IsCT1—when tested with *Bacillus cereus* show significant bacteria growth inhibition at concentrations lower than 10 μg/mL. However, increasing the concentration does not increase inhibition and bacterial growth reaches a plateau or increases slightly (Figure S2).

### 2.4. Hemolytic Activity

**Figure 2:**
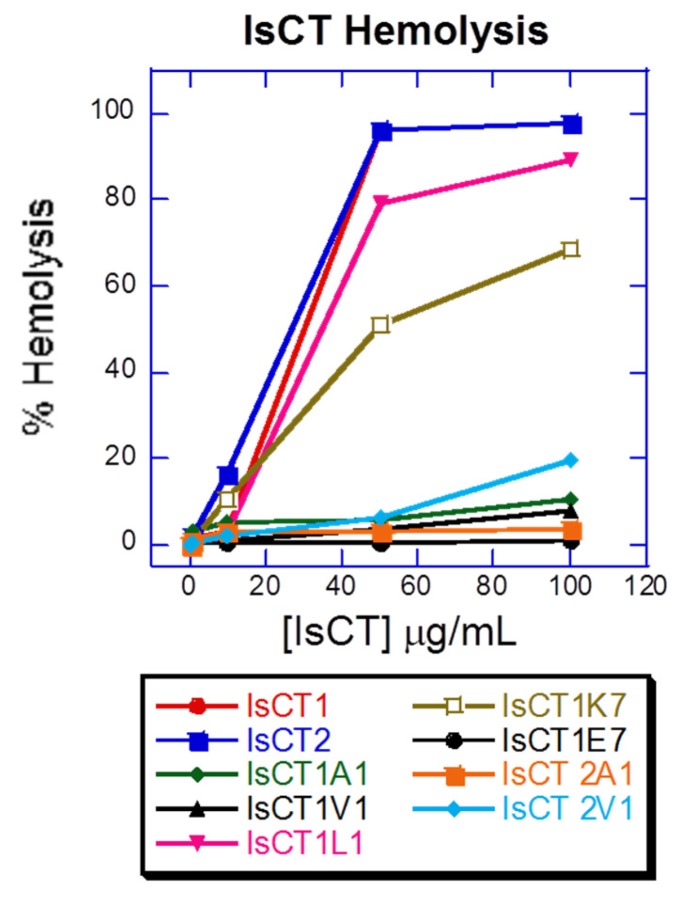
Hemolytic activity of peptide analogs.

The original peptides, along with the the leucine and lysine substituted analogs (IsCT1L1 and IsCT1K7), show the highest hemolytic activity. The other peptide analogs have negligible toxicity against erythrocytes under the conditions and concentrations tested, particularly the analog with a negative charge (IsCT1E7), which has no toxicity at all (Figure 2).

## 3. Discussion

Although recent studies propose novel mechanisms of action for antimicrobial peptides inside the bacterial cytoplasm [15], the most commonly accepted mechanism implies the binding of the peptides to the cell membrane surface which creates a mechanical disruption followed by the total destruction of the cell membranes [10]. The typical features for antimicrobial peptides are the formation of a well-defined secondary structure, usually a α-helix or a β-sheet, and the presence of both hydrophobic and hydrophilic regions creating an amphiphilic (also called amphipathic) nature necessary for the proposed mechanism of action of these antimicrobial peptides [20]. Though a more in-depth study of the structure these peptides and their interaction with the bacteria cell membranes will be necessary to fully correlate structure with activity, preliminary results with our synthetic seven analogs of the natural IsCT1 and IsCT2 peptides show they are able to form secondary α-helix structures. As mentioned, according to these results, it seems that an increase in size of the substituted amino acid (from alanine to valine to leucine) increases the percent helix, probably due to an increase in hydrophobicity with the introduction of more hydrophobic amino acids. The larger size of the amino acids seems not to affect helix structure. Changes in peptide net charge from +3 (IsCT1K7) to −1 (IsCT1E7) do not perturb the helical structure either.

Studies of bacterial inhibition carried out by Dai with IsCT1 and IsCT2 natural peptides show a range of MICs of 1 to 5 μg/mL for different strains of *S. aureus* and 10 to 25 μg/mL for two strains of *Bacillus* [21]. For Gram-negative bacteria, they show 5 to 120 μg/mL for two strains of *E. coli* but 100 or more than 200 μg/mL for other strains of Gram-negative bacteria (*Pseudomonas aeruginosa* and *Proteus mirabilis*). Bacterial inhibition studies by Kim with IsCT show MICs of 6 μg/mL for *E. coli* and 3 μg/mL for *S. aureus* and, for the IsCT1K7 analog, the reported concentrations are 3 and 1.5 μg/mL, respectively. Our results show MICs higher than the tested peptide concentrations of 100 μg/mL for all the tested bacteria except for *S. aureus* and *E. coli*. For the leucine substituted IsCTL1 analog, growth of *S. aureus* was inhibited at around 20–25 μg/mL while *E. coli* was inhibited at 15 and 25 μg/mL. As mentioned above, some analogs such as valine (IsCT1V1) or glutamic acid (IsCT1E7) substituted peptides show that bacteria growth is inhibited at low concentrations (10 μg/mL) followed by a leveling off or even an apparent increase in bacterial growth at higher concentrations. This phenomenon has been observed previously and a possible explanation is the formation of peptide aggregates [19].

It is also clear that the peptides that show higher bacteria growth inhibition—the natural IsCT1 and IsCT2, as well as the analogs IsCT1L1 and IsCT1K7—show the highest hemolytic activity. However other analogs, especially IsCT1E7 which has a net negative charge, though they do not appear to have a significant bacterial growth inhibition, do not have any apparent citotoxicity either under the tested concentrations and conditions. This result can be used for future design of similar antimicrobial peptides.

## 4. Materials and Methods

### 4.1. Reagents

All Fmoc-protected amino acids, the activating reagent 2-(1*H*-benzotriazol-1-yl)-1,1,3,3-tetramethyluronium hexafluorophosphate (HBTU), and Rink-Amide AM Resin (200–400 nm mesh) used for the manual solid phase peptide synthesis were purchased from Nova-Biochem. Dimethyl formamide (DMF), acetonitrile, diisopropyl ethyl amine, trifluoro acetic acid, and bacteria broth were purchased from Fisher Scientific to the purest quality and used without further purification. Bacteria were supplied by Rhodes College Department of Biology collection.

### 4.2. Peptide Synthesis and Purification

The synthesis and purification of the peptides was carried out as described previously [19]. Briefly, peptides were synthesized using manual solid-phase peptide synthesis with fluorenyloxycarbonyl (Fmoc) chemistry. Purification was carried out by reversed-phase high-performance liquid chromatography (RP-HPLC) with an acetonitrile/water gradient and 0.08% trifluoro acetic acid. Retention times range from 17 to 23 min (Table 1). Peptides were characterized by mass spectrometry. All peptides were dissolved in water to a concentration of 2 mg/mL (milligrams/milliliter) to form stock solutions and stored at −20 °C. Peptide solutions used for all assays were formed by diluting aliquots of stock solutions to the required μg/mL concentrations.

### 4.3. Characterization of Helical Structure

The mean residue ellipticities of the peptides were determined in water and in the presence of α-helix inducing solvent, 2,2,2-trifluoroethanol (TFE) at 50% in water by circular dichroism (CD). The mean residue molar ellipticity, [θ], is given in deg·cm^2^·dmol^−1^ and was calculated as described previously [19]. Values are the result of three independent experiments.

### 4.4. Bacteria Strains

Two Gram-positive bacteria: *Staphylococcus aureus* (ATCC# 29213) and *Bacillus cereus *(ATCC# 11778); and three Gram-negative bacteria: *Salmonella typhimurium* (ATCC# 14028), *Enterobacter aerogenes* (ATCC# 13048), and *Escherichia coli* (ATCC# 25922) were tested.

### 4.5. Measurement of Antibacterial Activity

Activity was determined by the standard liquid dilution method described previously [19]. All results are the average of three independent experiments. Briefly, cells were grown overnight at 37 °C in Luria Bertani broth and diluted in the same medium for assays. Bacteria were added to serial dilutions of the peptides (final concentrations of 1, 10, 50, and 100 μg/mL) and incubated at 37 °C for 24 h. Cell growth was measured by UV absorbance at 600 nm. For each bacterium, a solution containing no peptide (concentration 0 μg/mL) was processed under identical conditions and used as control for 100% bacterial growth. MICs were obtained from interpolation of 0% bacterial growth from plots. Results are the average of three independent experiments.

### 4.6. Measurement of Hemolytic Activity

Hemolytic activity was carried out as described previously [19]. Briefly, serial concentrations of peptides (final concentrations of 0 as blank, 1, 10, 50, and 100 μg/mL) were added to 1% human erythrocytes in 1× PBS buffer. The cell suspensions were incubated at 37 °C for 1 h and then centrifuged to remove unlysed erythrocytes. Released hemoglobin was determined spectrophotometrically at 416 nm. The control for 100% release of hemoglobin was a sample of 1% erythrocyte incubated in PBS with 1% TritonX-100. Results are the average of two independent experiments.

## 5. Conclusions

Our study with analogs of IsCT peptides found in scorpion venoms has shown that natural or wild type peptides have the highest bacterial growth inhibition compared to their synthetic analogs here studied. Of these, leucine substituted analog IsCT1L1, shows higher antibacterial activity but also toxicity similar to the wild type peptides. An increase in the net positive charge in IsCT1K7 or the net negative charge in IsCT1E7 does not improve the inhibition much. However, the fact that analog IsCT1E7 along with others have no apparent cytotoxicity for the concentrations tested is a promising starting point to design future potential antibacterial peptides.

## Figures and Tables

**Table 1:** Amino acid sequences, molecular masses, and HPLC retention times of IsCT peptides.

Peptide	Amino Acid Sequence ^a^	Molecular Mass		HPLC Retention Times	Net Charge
		Calculated	Observed	(min.)	
IsCT1	ILGKIWEGIKSLF-NH_2_	1502.9	1504.2	22.7	+1
IsCT2	IFGAIWNGIKSLF-NH_2_	1464.8	1466.0	20.5	+1
IsCT1A1	ILGK**A**WEG**A**KSLF-NH_2_	1418.7	1419.8	17.5	+1
IsCT1V1	ILGK**V**WEG**V**KSLF-NH_2_	1474.8	1476.0	19.0	+1
IsCT1L1	ILGK**L**WEG**L**KSLF-NH_2_	1502.9	1503.8	20.6	+1
IsCT1K7	ILGKIW**K**GIKSLF-NH_2_	1501.9	1502.1	20.0	+3
IsCT1E7	ILGKIW**E**GI**E**SLF-NH_2_	1503.8	1505.0	22.0	−1
IsCT2A1	ILGA**A**WNG**A**KSLF-NH_2_	1380.6	1380.8	19.3	+1
IsCT2V1	ILGA**V**WNG**V**KSLF-NH_2_	1436.7	1437.1	20.5	+1

^a^ Bold letters indicate the substituted amino acids.

**Table 2:** α-Helical content of IsCT peptides in water and 50% TFE.

Peptides	Water		50% TFE	
	[θ]_222_	% Helix	[θ]_222_	% Helix
IsCT1	−2046.57	Random	−11,484.85	30.2
IsCT2	−1242.43	Random	−9006.83	22.0
IsCT1A1	−1408.15	Random	−6602.05	14.1
IsCT1V1	−1722.88	Random	−8803.10	21.3
IsCT1L1	−1855.45	Random	−10,500.37	27.0
IsCT1K7	−1520.41	Random	−9533.00	23.8
IsCT1E7	−2561.86	1.0	−12,183.56	33.0
IsCT2A1	−89.92	Random	−3633.66	4.3
IsCT2V1	−793.14	Random	−6586.81	14.0

**Table 3:** MIC (μg/mL). Antibacterial activity of IsCT1 and IsCT2 analogs.

	MIC (μg/mL)				
	*S. aureus*	*B. cereus*	*S. typhimurium*	*E. aerogenes*	*E. coli*
IsCT1	50	>100	100	>100	50
IsCT2	50	100	100	>100	50
IsCT1A1	>100	>100	>100	>100	>100
IsCT1V1	>100	>100	>100	>100	>100
IsCT1L1	50	>100	100	>100	50
IsCT1K7	100	>100	100	>100	>100
IsCT1E7	>100	>100	>100	>100	>100
IsCT2A1	>100	>100	>100	>100	>100
IsCT2V1	>100	>100	>100	>100	>100

## Supplementary Materials

The following are available online at www.mdpi.com/2079-6382/6/3/13/s1, Figure S1: Circular Dichroism (CD) plots for IsCT peptides in water and 50% TFE, Figure S2: Bacteria inhibition with peptide analogues.
