# Supplementary material for: Antibacterial Activity and Toxicity of Analogs of Scorpion Venom IsCT Peptides
Source: Antibiotics (Basel). 2017 Jun 28;6(3):13. doi: 10.3390/antibiotics6030013 (PMC5617977; doi:10.3390/antibiotics6030013)
Supplement: Supplementary file 1 [file antibiotics-06-00013-s001.pdf]

Supplementary Materials: Antibacterial Activity and Toxicity of Analogs of Scorpion Venom IsCT Peptides

Roberto de la Salud Bea, Adam F. Petraglia, Michael R. Ascuitto and Quentin M. Buck

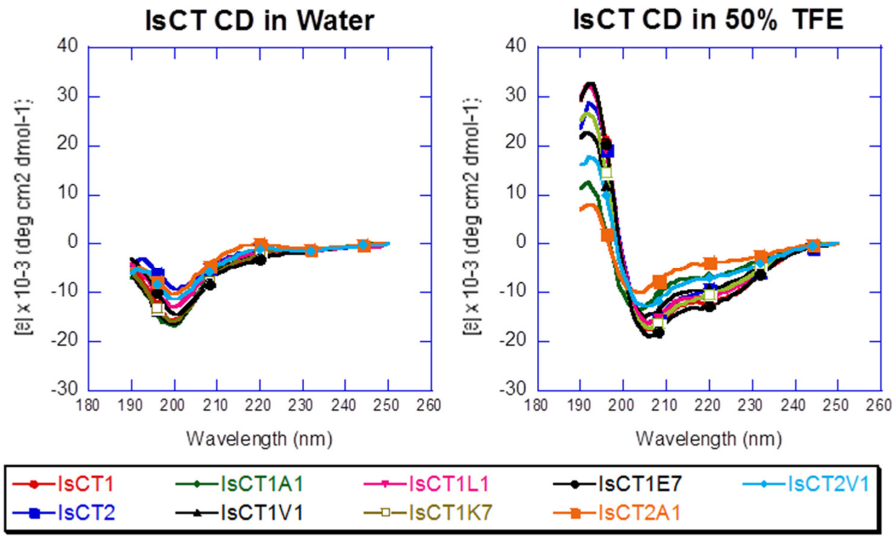

Figure S1. Circular Dichroism (CD) plots for IsCT peptides in water and 50% TFE.

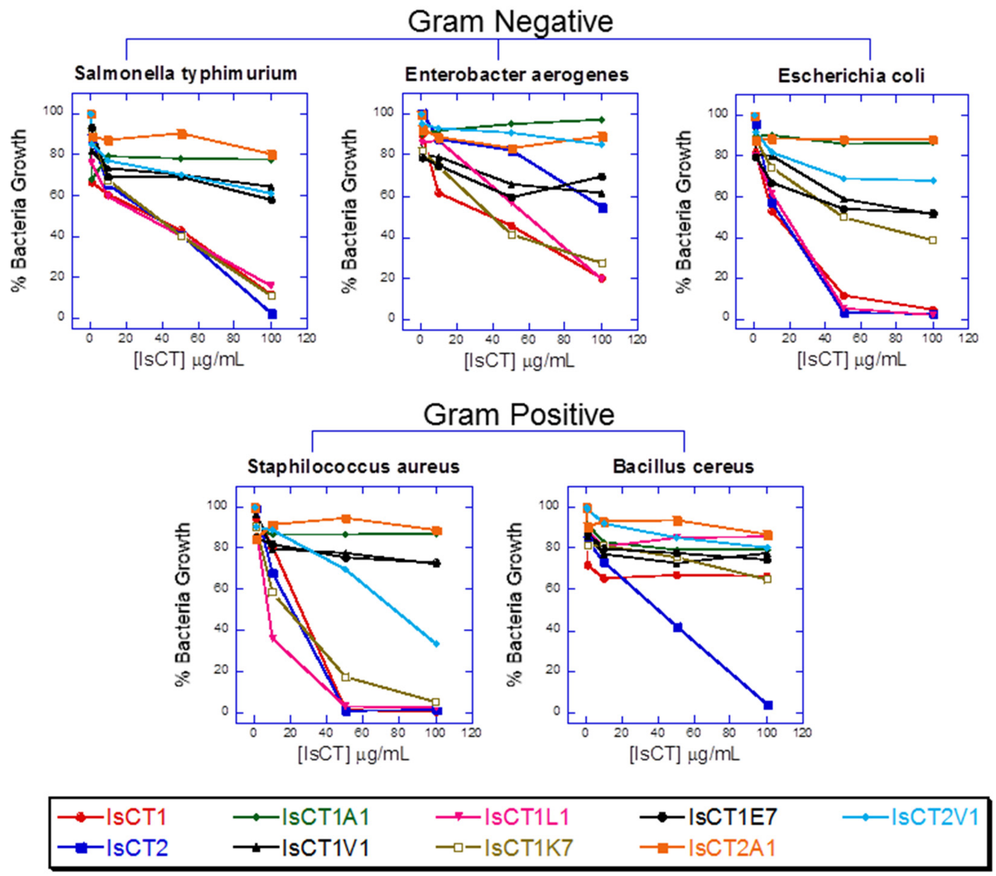

Figure S2. Bacteria inhibition with peptide analogs.

© 2017 by the authors; licensee MDPI, Basel, Switzerland. This article is an open access article distributed under the terms and conditions of the Creative Commons by Attribution (CC-BY) license (http://creativecommons.org/licenses/by/4.0/).
